# Supplementary material for: Discrete photoentrainment of mammalian central clock is regulated by bi-stable dynamic network in the suprachiasmatic nucleus
Source: Nat Commun. 2025 Apr 8;16:3331. doi: 10.1038/s41467-025-58661-1 (PMC11978930; doi:10.1038/s41467-025-58661-1)
Supplement: Supplementary file 1 — Supplementary Information [file 41467_2025_58661_MOESM1_ESM.pdf]

Supplementary Figures

Supplementary Fig. 1: Labeling cFos-positive neurons with the TRAP system.

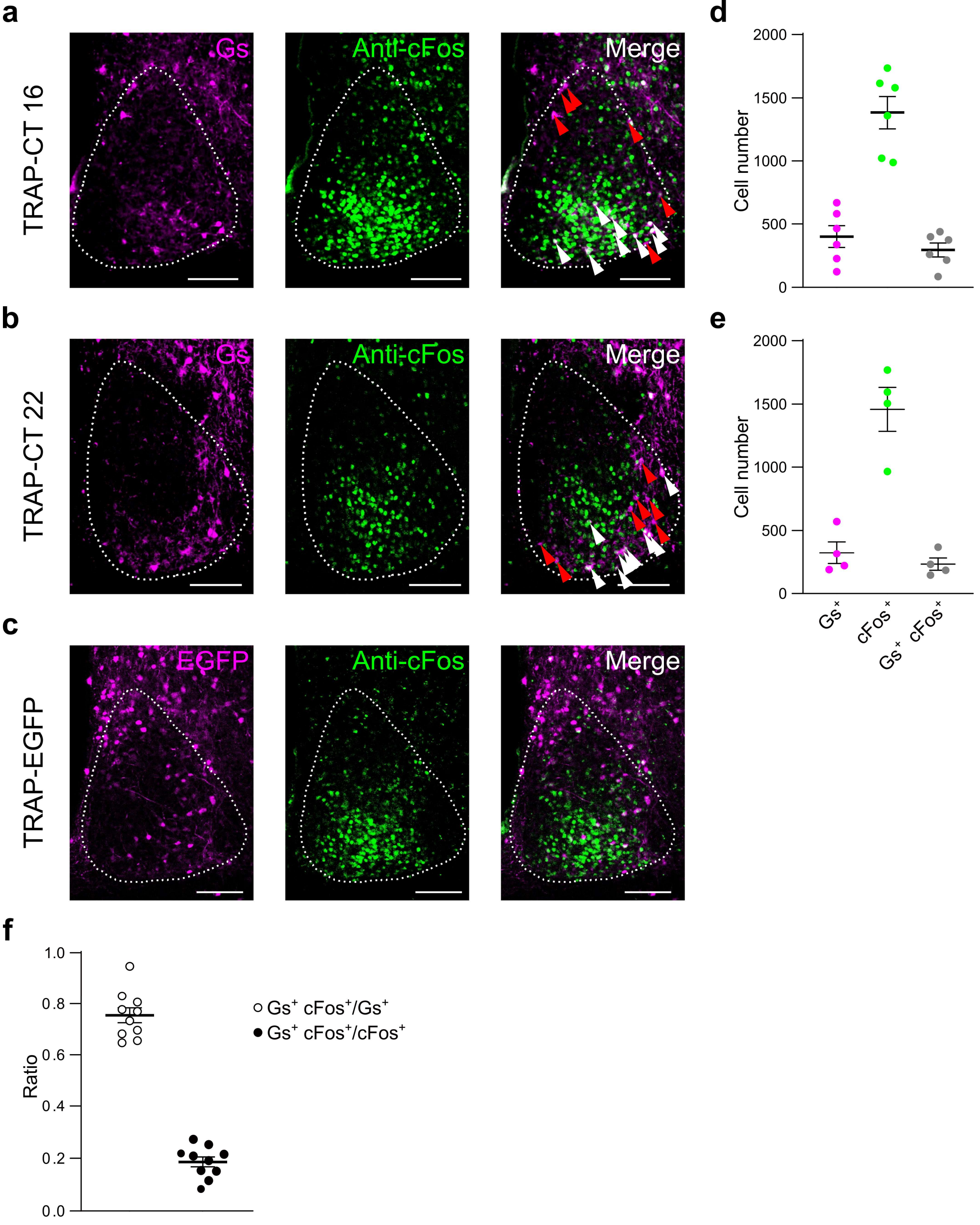

a. Representative image for DREADDs (rM3Ds)-expressing CT 16-trapped light response and cFos immune-positive SCN neurons for CT 16 light pulse. **b.** Representative image for DREADDs (rM3Ds)-expressing CT 22-trapped light response and cFos immune-positive SCN neurons for CT 22 light pulse. **c.** Representative image for EGFP control and cFos immune-positive SCN neurons for CT 16 light pulse. The dotted line depicts the SCN region derived by DAPI. Red arrows indicate only Gs-positive SCN neurons, while white arrows represent the double positive of Gs and anti-cFos SCN neurons. **d. & e.** Dot plots show cell numbers for Gs-expressing, anti-cFos, and double-positive SCN neurons from CT 16 and CT 22 TRAP mice. **f.** The dot plot shows the specificity and efficiency reflected by the colocalized ratio in the CT 16 and CT 22-trapped SCN neurons. Scale bars: 100 μm. n = 6 for d, n = 4 for e, and n = 10 for f. Error bars indicate means with SEM.

Supplementary Fig. 2: A light pulse produces a typical phase shift in TRAP mice.

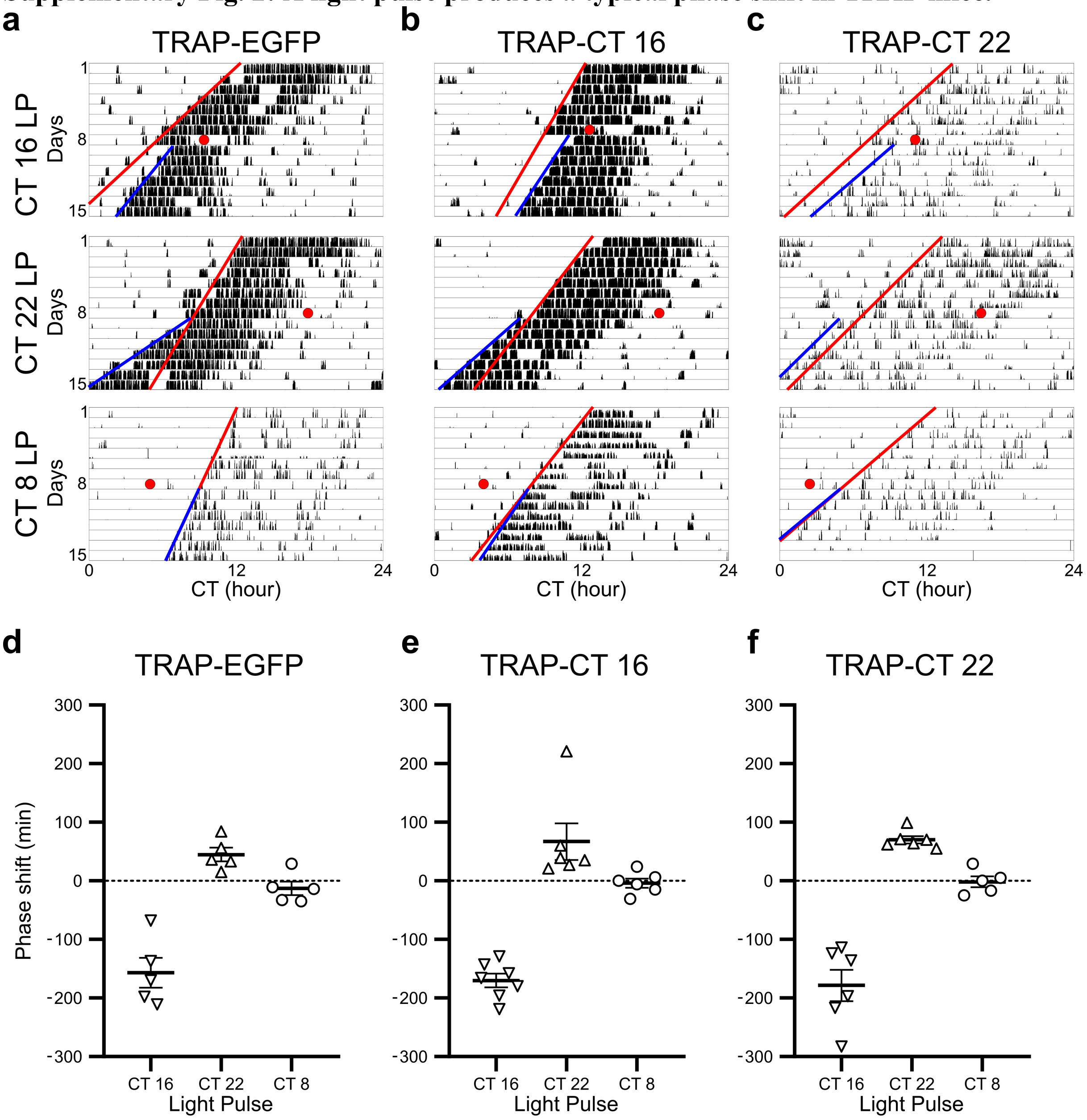

a. Representative actogram for light-induced phase shift in GFP-expressing TRAP mice. **b.** Representative actogram for DREADDs (rM3Ds)-expressing TRAP-CT 16 mice. c**.** Representative actogram for DREADDs (rM3Ds)-expressing TRAP-CT 22 mice. The red dots represent the time points of light pulse treatments, while the red line depicts an extended linear regression based on activity onsets before the treatment. The blue lines reflect the actual activity onsets following the light pulse treatment. **d.** Statistics of phase shift analysis for light-induced phase shift in GFP-expressing TRAP mice. n= 5. **e.** phase shift analysis for light-induced phase shift in DREADDs (rM3Ds)-expressing TRAP-CT 16 mice. n= 4. **f.** phase shift analysis for light-induced phase shift in DREADDs (rM3Ds)-expressing TRAP- CT22 mice. n = 5/5/5, 7/6/6, and 6/6/5 as CT 16/CT 22/CT 8 for d, e, and f respectively. Error bars indicate means with SEM.

Supplementary Fig. 3: Chemogenetic activation of TRAPed neurons in TRAP-CT16 mice produces a phase delay at CT 22 and CT 2.

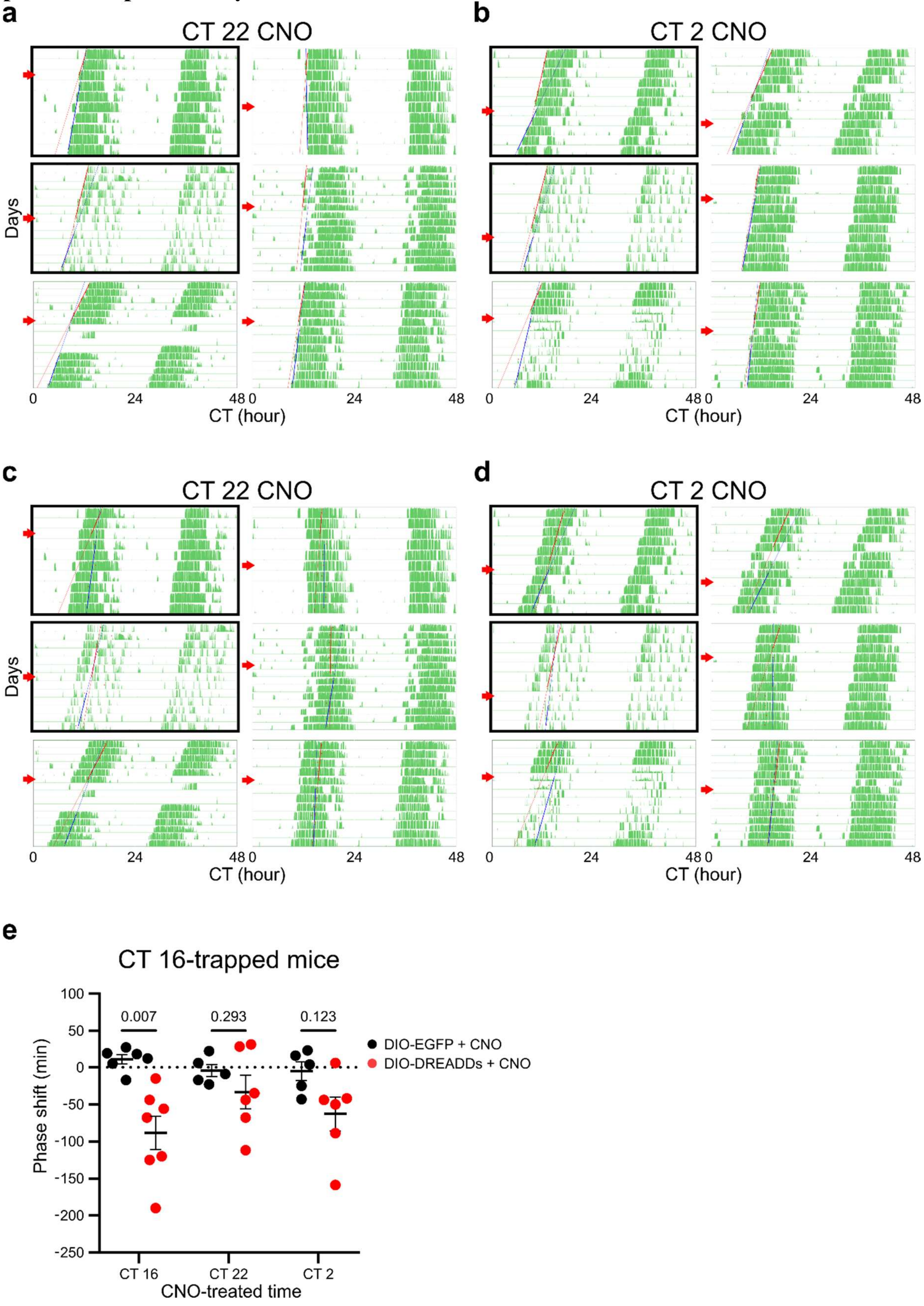

a-b. Double plot actograms for TRAP-CT16 mice injected with CNO at CT 22 or CT 2. Two heterozygous Fos-iCreER mice are indicated with thick outlines. Red lines are best-fit lines generated with activity onset before CNO injection. Blue lines are best-fit lines generated with activity onset after CNO injection. **c-d.** The same actograms as (a) and (b) marked with activity acrophase. **e.** Statistical analysis of CNO injection-induced phase shift calculated by acrophase from TRAP-CT16 and GFP control mice. Arrows indicate days of CNO injection. Error bars indicate mean with SEM. Numbers indicate p-values from Holm-Šídák multiple t-test. n = 6/7, 5/6, 5/6 as EGFP/DREADDs groups.

Supplementary Fig. 4: Vehicle injection control experiment in TRAP mice.

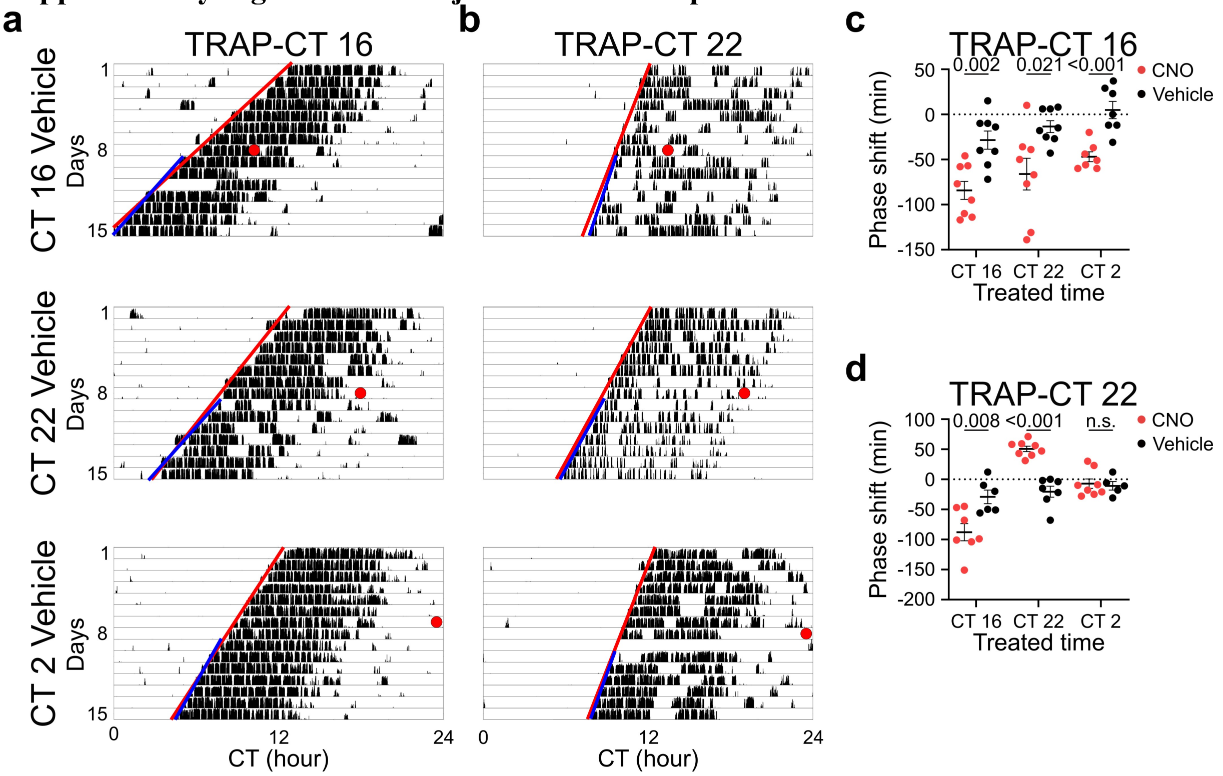

a. Representative actogram for DREADDs (rM3Ds)-expressing TRAP-CT 16 mice. **b.** Representative actogram for DREADDs (rM3Ds)-expressing TRAP-CT 22 mice. The red dots represent the time points of vehicle (saline) injection, while the red line depicts an extended linear regression based on activity onsets before the treatment. The blue lines reflect the actual activity onsets following treatment. **c.** Statistics of phase shift analysis for vehicle (saline) treatment in the TRAP-CT 16 mice. Here, phase shifts were calculated using onset with the best-fit line generated according to onsets from 5-7 days prior to injection. n = 8/8, 8/8, 7/7 for CNO/vehicle groups. **d.** Statistics of phase shift analysis for CNO and vehicle treatment in the TRAP-CT 22 mice. CNO group was the same as in main Figure 1g. n = 7/6, 8/7, 8/5 for CNO/vehicle groups. Numbers indicate p-values and n.s. indicates $p > 0.05$, Two-way ANOVA and Šídák post hoc test, vehicle group, Error bars indicate means with SEM. Data for CNO injection groups in d is the same as in main Figure 1g.

Supplementary Fig. 5: Chemogenetic activation of dark-TRAPed SCN neurons does not produce a phase shift at CT16.

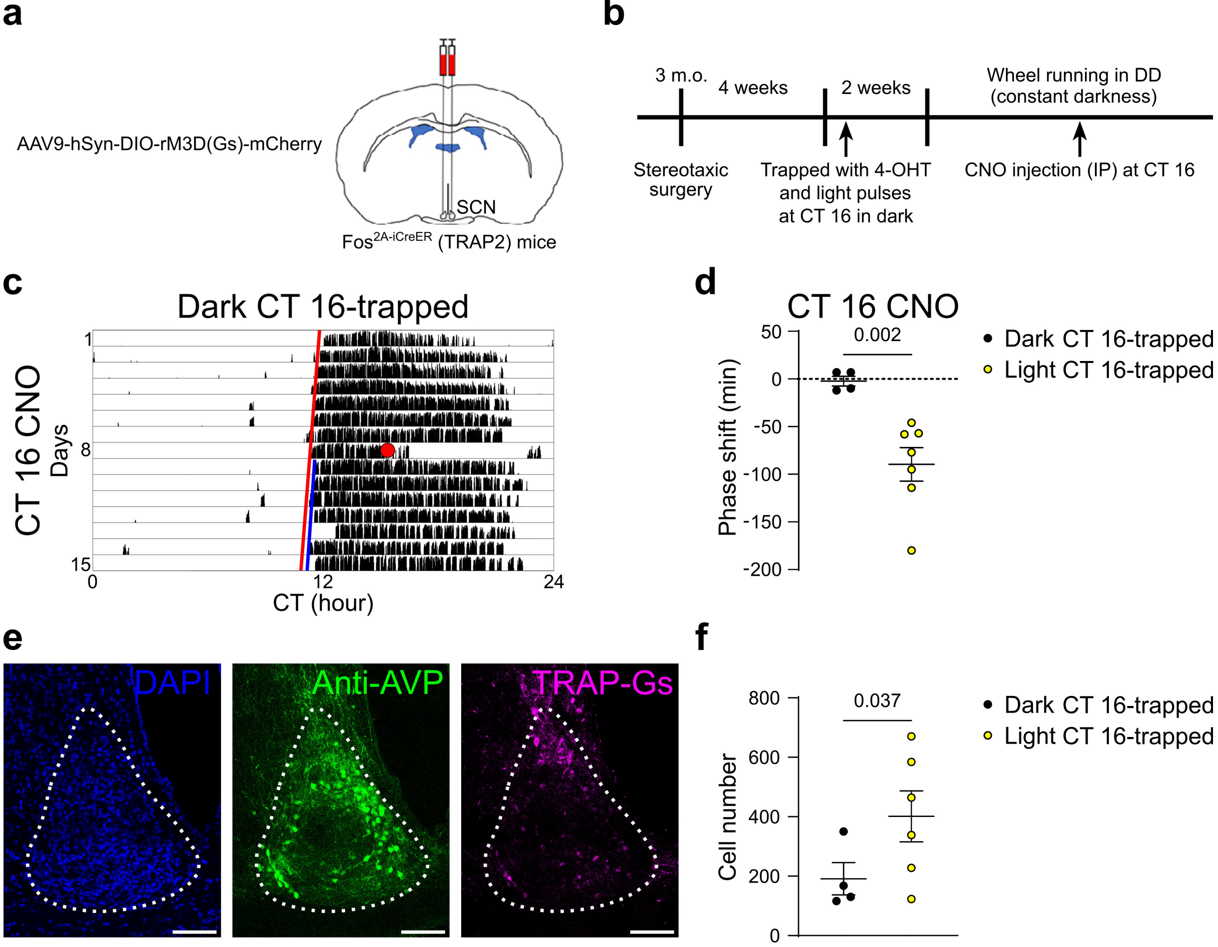

a. Experimental scheme for DREADDs bilateral SCN injection in the TRAP2 (Fos-iCreER) mice. **b.** Timetable for the experimental procedure in the CT 16 dark-trapped mice. **c.** Representative actogram for DREADDs (rM3Ds)-expressing CT 16 dark-trapped mice before and after CNO injection. The red dot represents the time points of CNO injection, while the red line depicts an extended linear regression based on activity onsets before the treatment. The blue lines reflect the actual activity onsets following treatment. **d.** Statistics of phase shift analysis for DREADDs (rM3Ds)-expressing CT 16 dark-trapped mice. **e.** Representative image for DREADDs (rM3Ds)-expressing CT 16 dark-trapped light response and anti-AVP immune-positive SCN neurons. **f.** Statistics of cell number for DREADDs (rM3Ds)-expressing CT16 dark-trapped mice. Numbers indicate p-value from unpaired two-tailed t-test, n = 4/7 and 4/6 as dark/light CT 16-trapped for d and f respectively. Error bars indicate means with SEM.

Supplementary Fig. 6: All identified ROIs in the SCN using GRIN lens two-photon imaging.

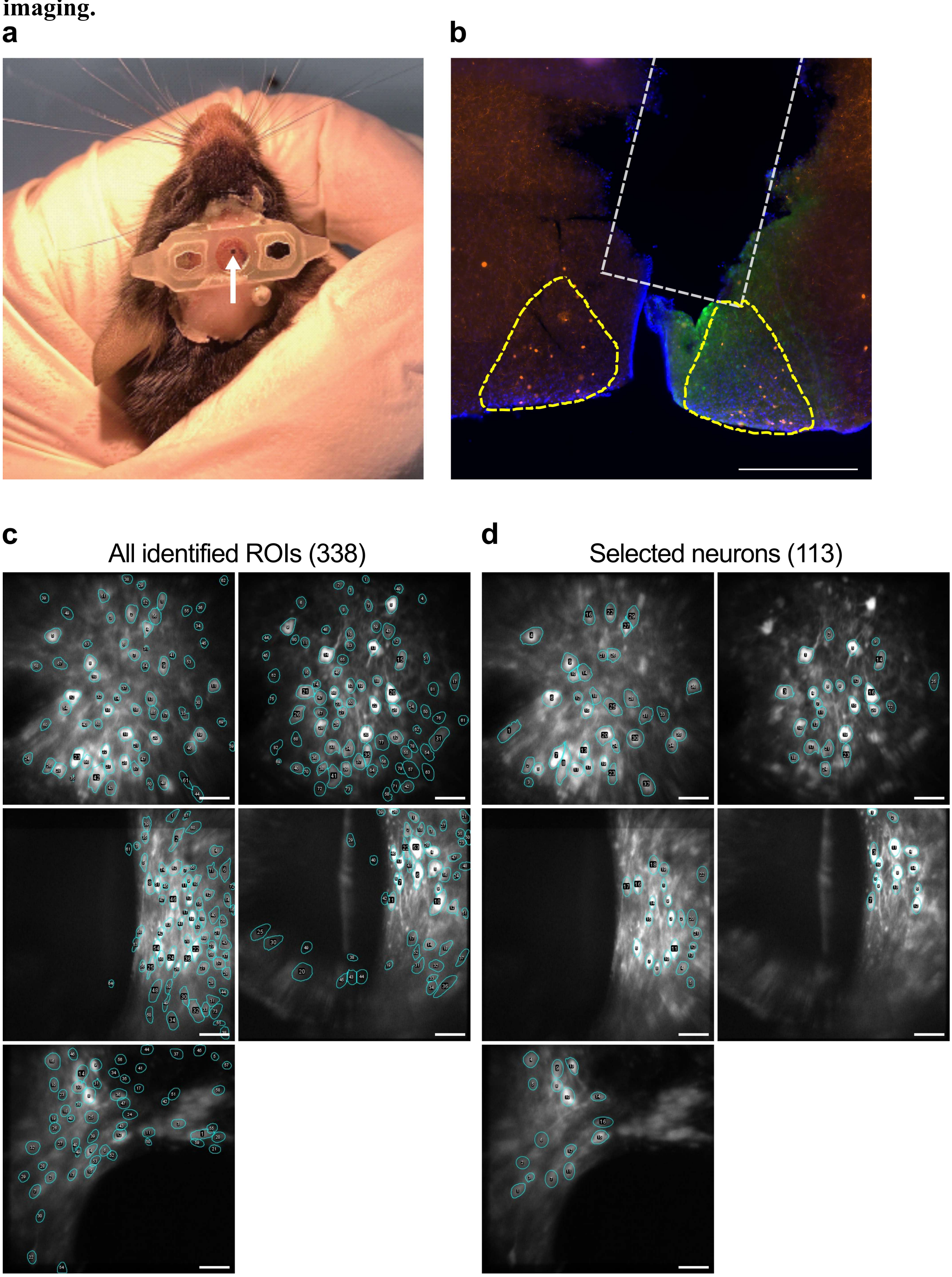

a. Photo of a mouse after GRIN endoscope implantation and head bar attachment. The head bars are to protect endoscopes from damage and to connect mice and the microscope. The arrowhead indicates GRIN endoscope. **b.** Immunofluorescence staining of the SCN (indicated by yellow outline) coronal section with the trajectory of an endoscope (indicated by white outline). Orange: tdTomato, green: anti-GFP, blue: DAPI. **c.** Representative images for all 5 recording focus planes and outlines indicate ROI identified at least once in 27 trials (341 in total). **d.** Representative images for all 5 focus planes and outlines indicate neurons identified in every trial (113 in total). Scale bars: 500 μm in (b) and 100 μm in (c) and (d).

Supplementary Fig. 7: Heat map of neuronal activity from all experiments.

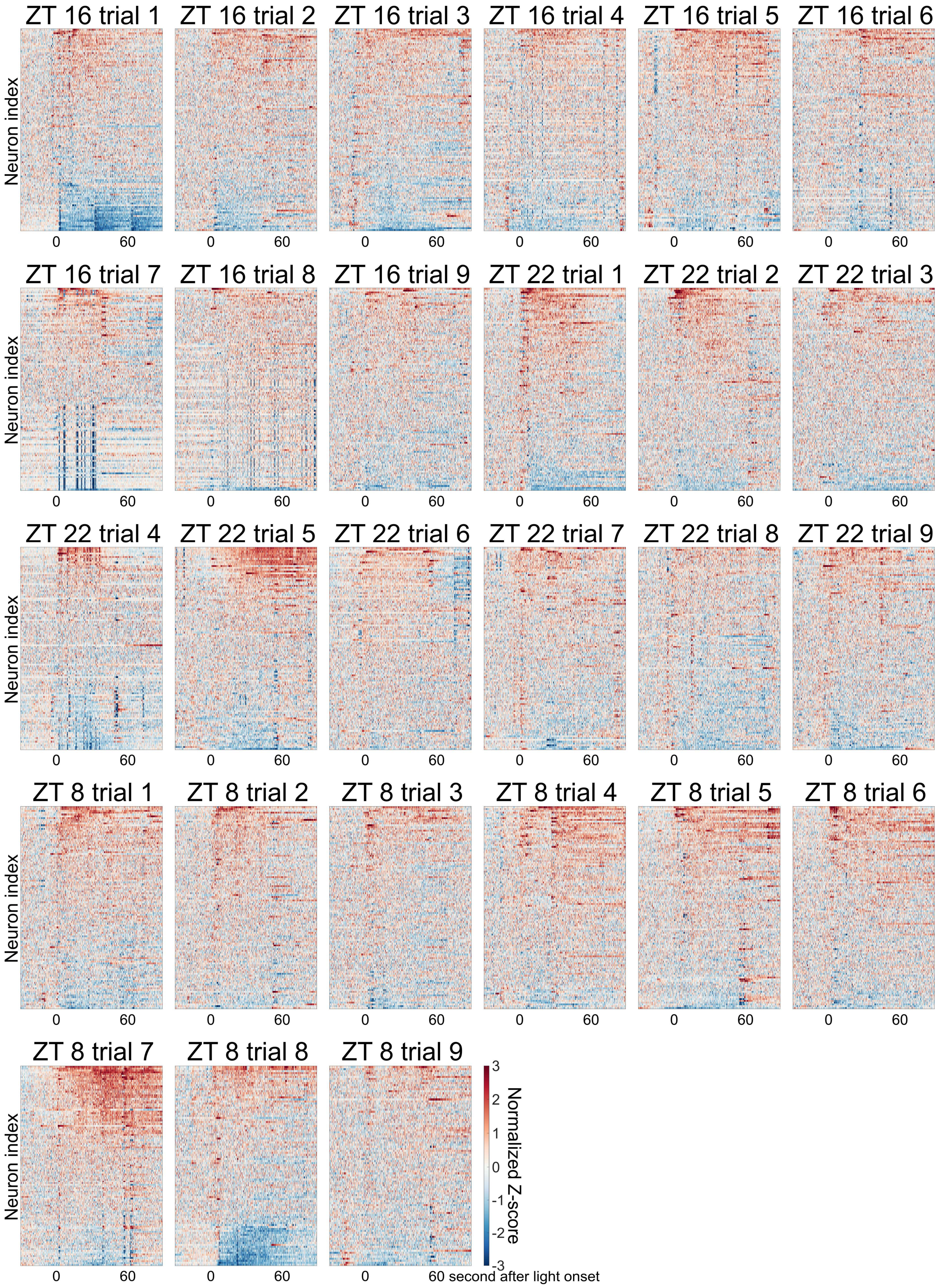

Heat map of normalized Z-score traces from 113 identified neurons in all 27 trials. Each trail is sorted with mean Z-score independently.

Supplementary Fig. 8: Correlation maps comparing peak times of VIP+ and VIP- neurons.

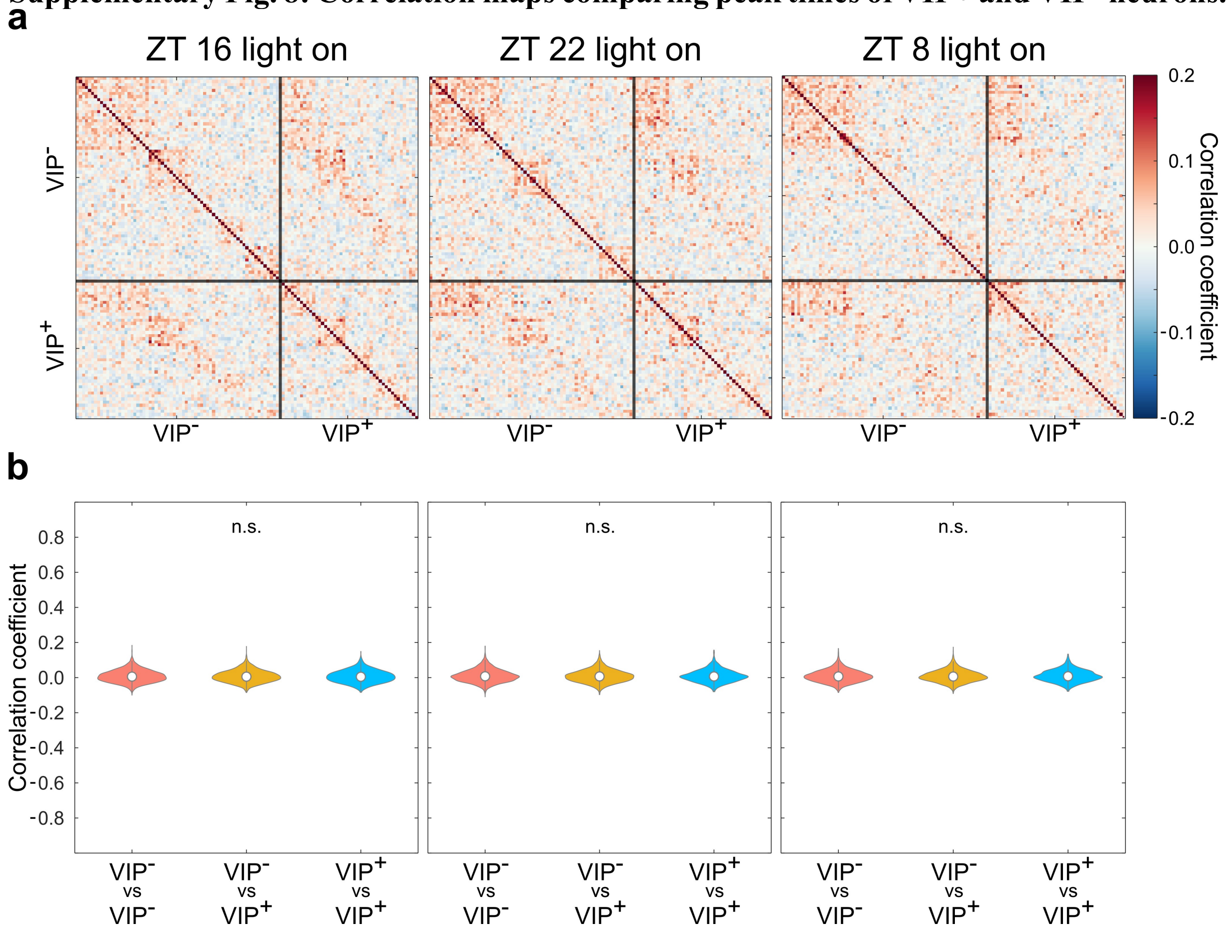

a. Each pixel on the map represents the Pearson correlation coefficient between peak times two neurons. **b.** Violin plots summarize the Pearson correlation coefficient of each comparison pair among cell types, including VIP$^-$ to VIP$^-$ (pink), VIP$^+$ to VIP$^-$ (yellow), and VIP$^+$ to VIP$^+$ (blue). n.s. indicates p-value > 0.05 from One-way ANOVA test.

Supplementary Fig. 9: Percentage of all clusters without significant differences between circadian times.

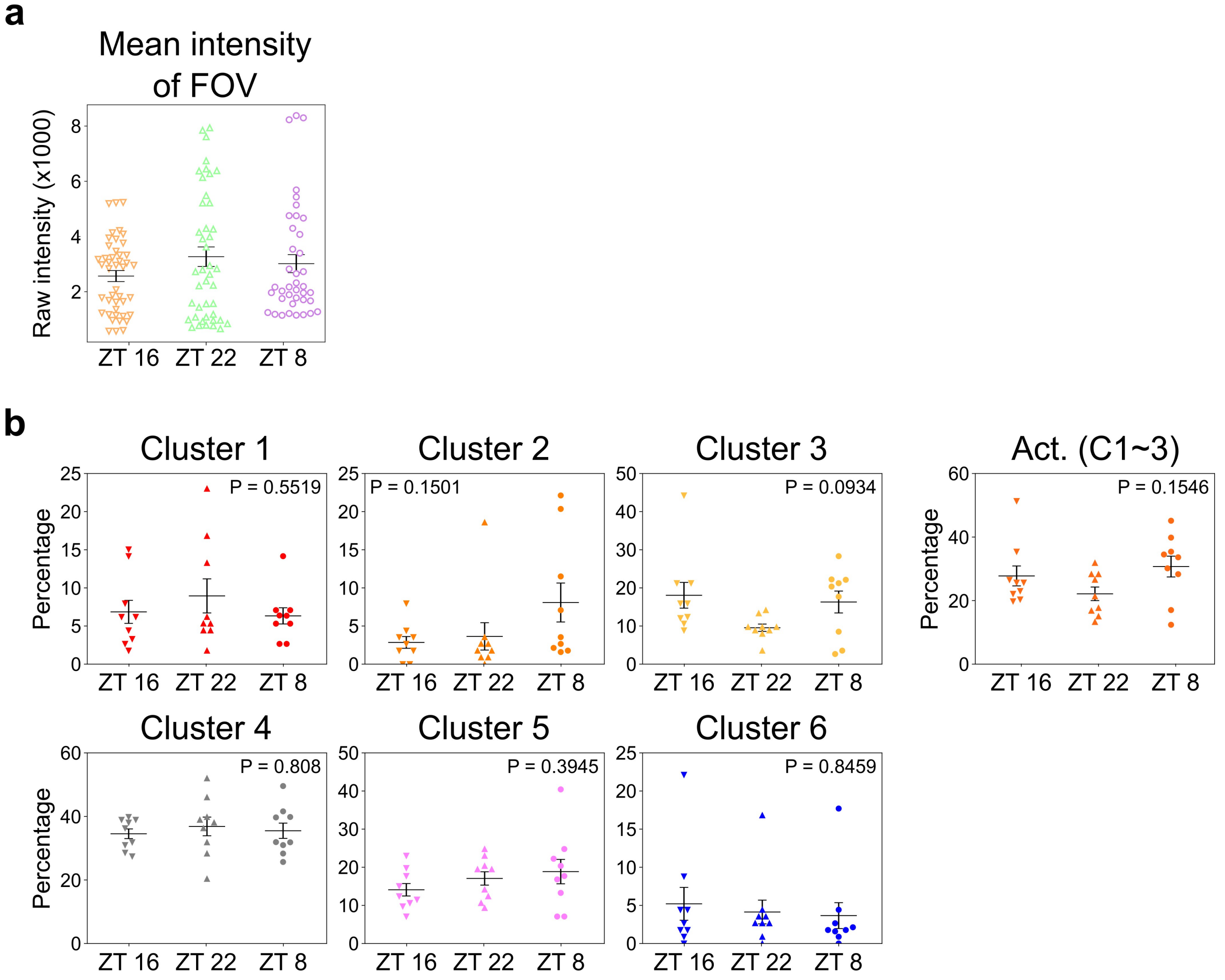

a. The mean of raw intensity of the fields of view (FOV). **b.** Comparing the percentage of neurons sorted into each cluster 1-6 between different ZTs and summation of activation clusters between different ZTs. P-values are calculated using one-way ANOVA and error bars indicate means with SEM. In a, n = 44, 43, 39 for ZT 16, ZT 22, ZT 8 respectively. In b, n = 9.

Supplementary Fig. 10: Highly connected neuron pairs from all recordings.

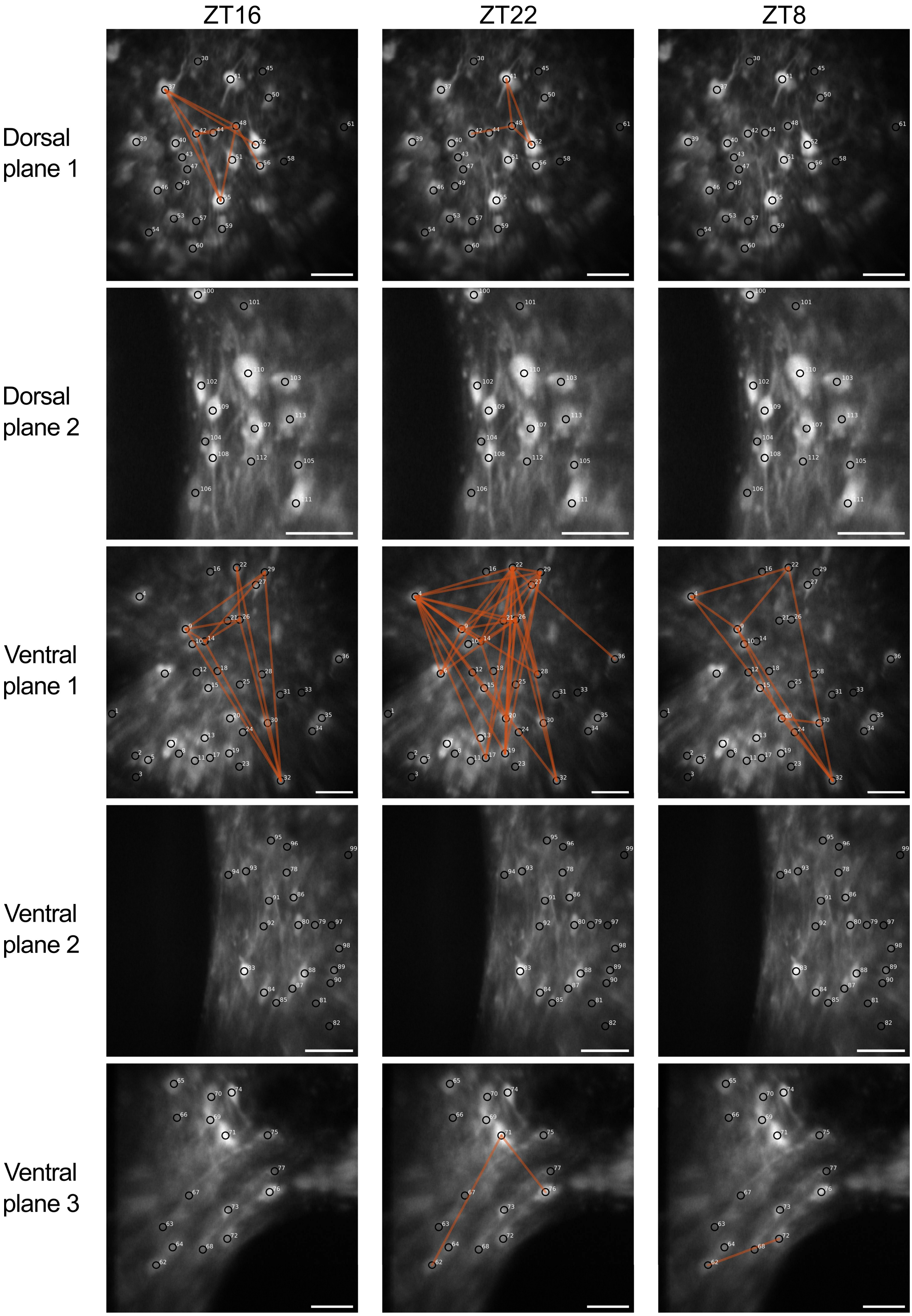

Highly correlated neuron pairs (average r from 9 repeats > 0.5) in all five layers from three time points are linked with orange lines. Scale bars: 100 µm.

Supplementary Fig. 11: Distinct calcium responses between group 1-3 neurons.

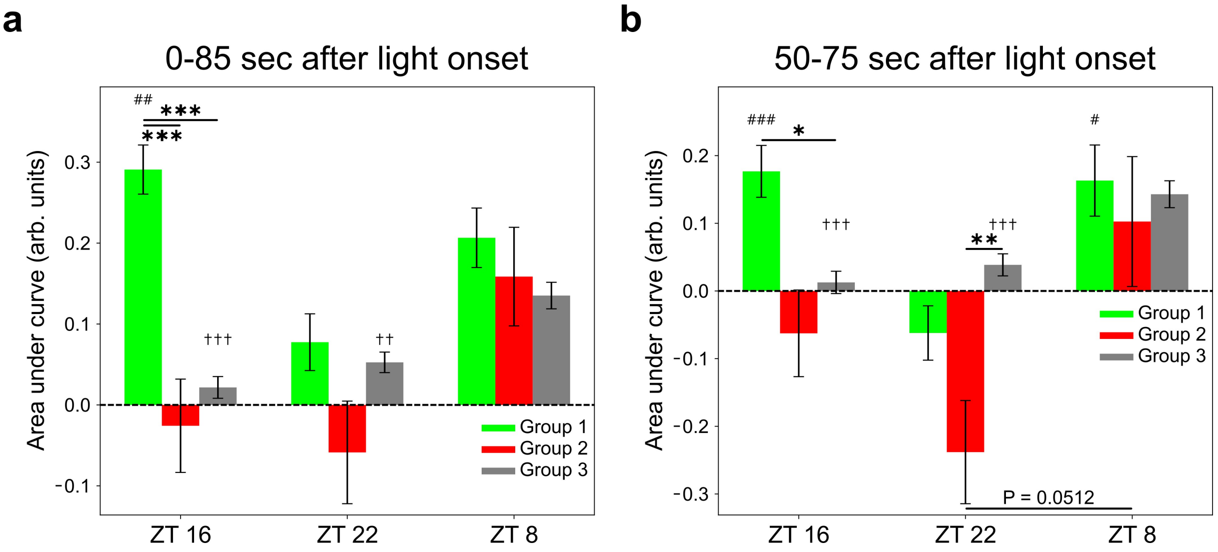

a-b. Area under the curve (AUC) calculated using normalized fluorescence Z-score from group 1-3 neurons between 0-85 sec under light stimulation (a) and 50-75 sec under light stimulation (b). Error bars indicate mean with SEM. Each data point represents AUC from one ROI in a single trial, n = 115, 44, 836, 114, 44, 821, 96, 37, and 768 from left to right respectively. P values were calculated with Two-way ANOVA and Tucky posthoc tests, * indicates significant differences between groups at the same ZT, † indicates significant differences compared to group 3 at ZT8, and # indicates significant differences compared to group 1 at ZT22. One to three markers indicate $p < 0.05$, $p < 0.01$, and $p < 0.001$ respectively.

Supplementary Fig. 12: Most group 3 neurons display a significant difference in variance after light exposure compared to baseline.

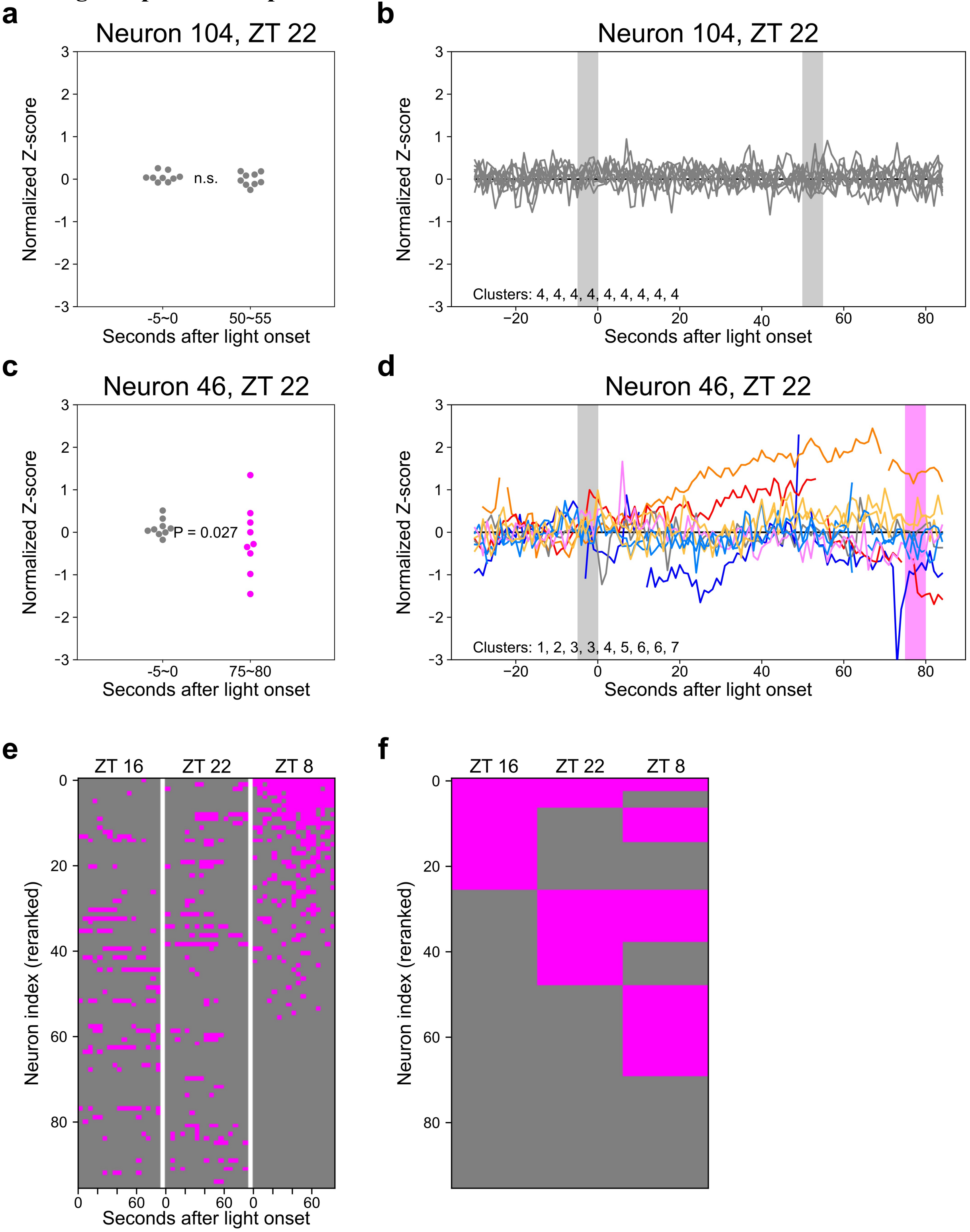

a & c. Representative neurons' normalized Z-scores from nine trials showing the baseline GCaMP response (5-second average) before light onset compared with the GCaMP response 50 and 75 seconds after light onset. P-value is calculated using Levene's test. n = 9. **b & d.** The full traces of nine trials for representative neurons. Each color represents a different light response cluster. a and b depict a representative group 3 neuron whose post-onset variances are not significantly greater than the pre-onset baseline. c & d illustrate another representative group 3 neuron with certain 5-second bins post-onset exhibiting significantly increased variance compared to the pre-onset baseline. **e.** The composite plot from group 3 neurons where significantly increased post-onset variances are highlighted in magenta. Levene's test for equality of variances, $p < 0.05$. **f.** Group 3 neurons are marked in magenta if at least one bin demonstrates significant variance in Levene's test. This analysis indicates that 69 of the group 3 neurons (72.6%) display high variation amount trails at the same ZTs.
