# Supplementary material for: Discrete photoentrainment of mammalian central clock is regulated by bi-stable dynamic network in the suprachiasmatic nucleus
Source: Nat Commun. 2025 Apr 8;16:3331. doi: 10.1038/s41467-025-58661-1 (PMC11978930; doi:10.1038/s41467-025-58661-1)
Supplement: Supplementary file 2 — Reporting Summary [file 41467_2025_58661_MOESM2_ESM.pdf]

nature portfolio

Corresponding author(s): ShihKuo Chen

Last updated by author(s): Feb 25, 2025

Reporting Summary

Nature Portfolio wishes to improve the reproducibility of the work that we publish. This form provides structure for consistency and transparency in reporting. For further information on Nature Portfolio policies, see our Editorial Policies and the Editorial Policy Checklist.

Statistics

For all statistical analyses, confirm that the following items are present in the figure legend, table legend, main text, or Methods section.

n/a	Confirmed	
☐	☒	The exact sample size (*n*) for each experimental group/condition, given as a discrete number and unit of measurement
☐	☒	A statement on whether measurements were taken from distinct samples or whether the same sample was measured repeatedly
☐	☒	The statistical test(s) used AND whether they are one- or two-sided<br>*Only common tests should be described solely by name; describe more complex techniques in the Methods section.*
☒	☐	A description of all covariates tested
☐	☒	A description of any assumptions or corrections, such as tests of normality and adjustment for multiple comparisons
☐	☒	A full description of the statistical parameters including central tendency (e.g. means) or other basic estimates (e.g. regression coefficient) AND variation (e.g. standard deviation) or associated estimates of uncertainty (e.g. confidence intervals)
☐	☒	For null hypothesis testing, the test statistic (e.g. *F*, *t*, *r*) with confidence intervals, effect sizes, degrees of freedom and *P* value noted<br>*Give P values as exact values whenever suitable.*
☒	☐	For Bayesian analysis, information on the choice of priors and Markov chain Monte Carlo settings
☒	☐	For hierarchical and complex designs, identification of the appropriate level for tests and full reporting of outcomes
☐	☒	Estimates of effect sizes (e.g. Cohen's *d*, Pearson's *r*), indicating how they were calculated

Our web collection on statistics for biologists contains articles on many of the points above.

Software and code

Policy information about availability of computer code

Data collection	Commercial software for Two-photon data collection, JadeBio, Southport. Commercial software for wheel running activity recording, Clocklab, Actimetrics.
Data analysis	Customized code were used for two-photon image motion correction. For ROI detection, Cellpose algorithm from GitHub were used. For wheel running activity analysis we used commercial software, Clocklab, Actimetrics. Statistical analysis were performed using Prism, GraphPad software, Python with scikit-learn, and MATLAB, The MathWorks.

For manuscripts utilizing custom algorithms or software that are central to the research but not yet described in published literature, software must be made available to editors and reviewers. We strongly encourage code deposition in a community repository (e.g. GitHub). See the Nature Portfolio guidelines for submitting code & software for further information.

Data

Policy information about availability of data

All manuscripts must include a data availability statement. This statement should provide the following information, where applicable:

- Accession codes, unique identifiers, or web links for publicly available datasets
- A description of any restrictions on data availability
- For clinical datasets or third party data, please ensure that the statement adheres to our policy

The data that support the findings of this study are included in the manuscript and source data files

April 2023

Research involving human participants, their data, or biological material

Policy information about studies with human participants or human data. See also policy information about sex, gender (identity/presentation), and sexual orientation and race, ethnicity and racism.

Reporting on sex and gender	n/a
Reporting on race, ethnicity, or other socially relevant groupings	n/a
Population characteristics	n/a
Recruitment	n/a
Ethics oversight	n/a

Note that full information on the approval of the study protocol must also be provided in the manuscript.

Field-specific reporting

Please select the one below that is the best fit for your research. If you are not sure, read the appropriate sections before making your selection.

☒ Life sciences ☐ Behavioural & social sciences ☐ Ecological, evolutionary & environmental sciences

For a reference copy of the document with all sections, see nature.com/documents/nr-reporting-summary-flat.pdf

Life sciences study design

All studies must disclose on these points even when the disclosure is negative.

Sample size	Sample size is determined according to previous literature on the similar research field.
Data exclusions	There is no data exclusion for current study except specifically stated in the main text.
Replication	For two-photon in vivo recording, 3 independent animals were used for replication. Data were pooled for analysis. For wheel running experiments, data were collected individually as replication.
Randomization	Animals were randomly allocated into different treatments.
Blinding	Data analysis was carried out using double blind analysis.

Reporting for specific materials, systems and methods

We require information from authors about some types of materials, experimental systems and methods used in many studies. Here, indicate whether each material, system or method listed is relevant to your study. If you are not sure if a list item applies to your research, read the appropriate section before selecting a response.

Materials & experimental systems

n/a	Involved in the study	
☐	☒	Antibodies
☒	☐	Eukaryotic cell lines
☒	☐	Palaeontology and archaeology
☐	☒	Animals and other organisms
☒	☐	Clinical data
☒	☐	Dual use research of concern
☒	☐	Plants

Methods

n/a	Involved in the study	
☒	☐	ChIP-seq
☒	☐	Flow cytometry
☒	☐	MRI-based neuroimaging

Antibodies

Antibodies used	anti-cFos mouse monoclonal antibody (1:1000, Abcam, cat#ab208942, lot#GR3454884-1), anti-vasopressin rabbit antibody (1:1000, Immunostar, cat#20069, product ID 20069), and anti-GFP chicken antibody (1:1000, Abcam, cat#ab13970, lot#GR89472-8)

Validation	Antibodies were all validated by production companies as stated on their website.

Animals and other research organisms

Policy information about studies involving animals; ARRIVE guidelines recommended for reporting animal research, and Sex and Gender in Research

Laboratory animals	All mice were kept under C57BL/6 background. Transgenic mice strain #031628, #007914, and #030323 were obtained from Jackson Lab, USA. Ages of mice for individual experiments were reported in method section.
Wild animals	This study did not involve wild animals.
Reporting on sex	For in vivo recording, we recorded from two male and one female mice. For wheel running activity, both male and female were used.
Field-collected samples	This study did not involve sample collected in the field.
Ethics oversight	Our research protocols were approved by National Taiwan University IACUC with protocol numbers: NTU107-EL-00169 and NTU-110-EL-00057.

Note that full information on the approval of the study protocol must also be provided in the manuscript.

Plants

Seed stocks	n/a
Novel plant genotypes	n/a
Authentication	n/a
